# Two neuronal groups for NaCl with differential taste response properties and topographical distributions in the rat parabrachial nucleus

**DOI:** 10.14814/phy2.14443

**Published:** 2020-05-22

**Authors:** Tatsuko Yokota, Nubuo Katakura, Takumi Morita, Tomoko Matsunaga, Katsunari Hiraba

**Affiliations:** ^1^ Department of Physiology School of Dentistry Aichi‐Gakuin University Nagoya Japan

**Keywords:** brachium conjunctivum, parabrachial nucleus, rostral nucleus of the solitary tract, taste response, topographical distribution

## Abstract

It is crucial for animals to discriminate between palatable (safe) and aversive (toxic) tastants. The mechanisms underlying neuronal discrimination of taste stimuli remain unclear. We examined relations between taste response properties (spike counts, response duration, and coefficient of variation [CV]) and location of taste‐sensitive neurons in the pontine parabrachial nucleus (PBN). Extracellular single units’ activity in the PBN of Wistar rats was recorded using multibarrel glass micropipettes under urethane anesthesia. Forty taste‐sensitive neurons were classified as NaCl (N)‐best (*n* = 15), NaCl/HCl (NH)‐best (*n* = 14), HCl (H)‐best (*n* = 8), and sucrose (S)‐best (*n* = 3) neurons. The net response to NaCl (15.2 ± 2.3 spikes/s) among the N‐best neurons was significantly larger than that among the NH‐best (4.5 ± 0.8 spikes/s) neurons. The response duration (4.5 ± 0.2 s) of the N‐best neurons to NaCl was significantly longer than that of the NH‐best (2.2 ± 0.3 s) neurons. These differences in the spike counts and the response durations between the two neuronal types in the PBN were similar to that previously reported in the rostral nucleus of the solitary tract (rNST). The CVs in the N‐best and the NH‐best neurons were significantly smaller in the PBN than those in the rNST. Histologically, most N‐best neurons (12/13, 92%) were localized to the medial region, while NH‐best neurons (11/13, 85%) were primarily found within the brachium conjunctivum. These results suggest that NaCl‐specific taste information is transmitted by two distinct neuronal groups (N‐best and NH‐best), with different taste properties and locations within rNST to PBN tractography. Future studies on the higher order nuclei for taste could reveal more palatable and aversive taste pathways.

## Graphical abstract

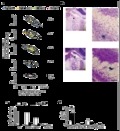

## 1 INTRODUCTION

Taste‐topic map in the cerebral gustatory cortex (Accolla, Bathellier, Petersen, & Carleton, 2007; Chen, Gabitto, Peng, Ryba, & Zuker, 2011; Peng et al., 2015; Yamamoto, Yuyama, Kato, & Kawamura, 1985) and the rostral nucleus of the solitary tract (rNST), a first‐order taste relay (Yokota, Eguchi, & Hiraba, 2014), implied topographical segregations for taste‐sensitive neurons in the pontine parabrachial nucleus (PBN), a second‐order taste relay. However, recent studies have reported non‐taste‐topic map in the gustatory cortex (Fletcher, Ogg, Lu, Ogg, & Boughter, 2017; Levitan et al., 2019). The PBN contains medial and lateral regions that surround the superior cerebellar peduncle, known as the brachium conjunctivum (BC). These regions are involved in multiple functions, including conveying taste information, gastrointestinal signals, respiratory cycles, and regulating cardiovascular function. Each of these functions is hypothesized to be segregated into distinct regions with some overlap in the PBN (Baird, Travers, & Travers, 2001; Cohen, 1971; Hayward & Felder, 1995; Herbert, Moga, & Saper, 1990; Karimnamazi, Travers, & Travers, 2002; Lara et al., 2002). Topographical distributions between different types of taste‐sensitive neurons were reported. For instance, HCl‐best (H‐best) (lateral and rostrally positioned) and NaCl‐best (N‐best) (caudal and medial portions) neurons are segregated in hamsters (Van Buskirk & Smith, 1981), rats (Ogawa, Hayama, & Ito, 1984), and sucrose‐best (S‐best) (medial and BC) and N‐best (lateral portion) in mice (Tokita & Boughter, 2016). However, others have reported a lack of topographical distribution in rats (Travers & Geran, 2009). In the rNST, we reported that N‐best neurons could be distinguished from NaCl/HCl (NH)‐best neurons not only based on their response properties but also based on the more rostral distribution of NH‐best neurons (Yokota et al., 2014). Given this, it is possible that the N‐best and NH‐best neurons in the PBN may be topographically distinct and may project to the PBN via distinct tracts. Taste information for palatable and aversive salts may be transmitted distinctly by N‐best and NH‐best neurons, respectively, because NaCl‐specific taste receptor cells (TRCs) responded to palatable (low) concentration of NaCl, and TRCs broadly tuned to NaCl and KCl responded only to aversive (high) concentration of NaCl (Chandrashekar et al., 2010).

In the present study, we investigated the relationship between response properties (spike count, response duration, and coefficient of variation [CV]) and locations of taste‐sensitive neurons in the PBN.

## 2 MATERIALS AND METHODS

### 2.1 Subjects

Thirty‐one adult male Wistar rats between 8 and 16 weeks of age (body weight: 290–450 g) were used to record PBN neural activity.

All experiments were performed in accordance with the National Institutes of Health's Guide for the Care and Use of Laboratory Animals and were approved by the Aichi‐Gakuin University's Animal Care and Use Committee. The animals were kept on a 12:12‐hr light:dark cycle with ad libitum access to food and water.

### 2.2 Surgical protocol

Animals were initially anesthetized with a mixture of urethane (0.6 g/kg, *i.p*.) and pentobarbital sodium (40 mg/kg, *i.p*.). The incision site was anesthetized using a local anesthetic (lidocaine hydrochloride; 12 mg/kg) injected into the animal's skin. After a tracheotomy for airway maintenance, the animals were restrained in a stereotaxic apparatus such that bregma and lambda were level. A craniotomy of the parietal bone (a 3 × 3‐mm section) was performed over the cerebrum or the cerebellum and a microelectrode was inserted into the PBN. Animals were artificially ventilated to maintain 4% end‐tidal Pco_2_. Electrocardiograms were monitored continuously and rectal temperature was maintained at approximately 37°C with the use of a heating pad. During recording, anesthesia was maintained with urethane (70 mg kg^−1^ h^−1^, *i.p*.), while muscle tone was decreased with gallamine triethiodide (90 mg kg^−1^ h^−1^, *i.p*.), which can be used to synchronize animals’ respiratory rhythms to that of an artificial ventilator. Taste responses were steadied for recordings as shown on raster displays in Figure 5. At the end of the experiment, the animals were euthanized by an overdose of pentobarbital sodium (120 mg/kg, *i.p*.).

### 2.3 Taste stimulation

The test solutions (25°C–26°C), which were applied into the oral cavity at a flow rate of 1.1–1.3 ml/s, were 0.2 M NaCl (N), 0.25 M sucrose (S), 0.04 M HCl (H), and 0.005 M quinine‐HCl (Q). To stimulate the anterior and posterior tongue and the oral cavity, the tongue was pulled anteriorly and mounted on the remaining sections of the lower jaw incisors, cut along their heights 1 mm above the gum. The stimulation nozzle was placed near the lingual tip. The solutions initially touched the soft palate, filled the oral cavity, and then drained through the lingual tip. Different test solutions were delivered successively in random order through solenoid valves that were controlled by a computer program written by custom scripts in LabVIEW (National Instrument). Operating signals sent from the computer to the solenoids were used as stimulus signals. Each test solution was applied for 6 s, followed by rinsing with distilled, deionized water for 14 s subsequent to pause for 2 s. Seven applications (trials) were performed with each solution at 2‐s pause between trials. To minimize the effects of solution temperature, data from the first two trials were not included in further analyses.

### 2.4 Estimation of taste response properties

**FIGURE 1:**
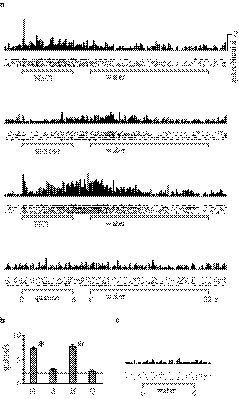
An example of taste‐sensitive neuron stimulation. (a) Mean peri‐stimulus time course histograms (100‐msec bin) are characterized by distinct tastant response patterns. The gray horizontal lines indicate the taste stimulation period (6 s) and rinsing water administration period (14 s). Spike densities are shown as raster plots (1‐msec bins, five trials) below histograms. (b) Asterisks show significant differences between mean spike activities in response to each solution: NaCl (N), sucrose (S), HCl (H), and quinine (Q), and the final 3 s of rinsing water administration (horizontal line), per Student's *t*‐test (*p* < .05). (c) The mean peri‐stimulus time course histograms to repetitive applications (five trials) of water only

For each taste stimulus, the number of spikes elicited during each stimulation period (6 s) was counted and averaged over five trials. The mean water response was calculated by averaging the number of spikes during the last 3 s of each 14‐s water response session. Neuronal excitability then declined as the neuron returned to its resting state in the last 3 s of water application (Figure 1a). To obtain the net taste response unaffected by somatosensory or thermal aspects of the test solutions, a mean response per second to the last 3 s of water application was subtracted from the mean number of spikes per second measured during the 6 s of taste stimulus. Significant differences in taste responses were examined via one‐way ANOVA for spike counts among the four tastants and distilled water for five trials (Figure 1b). *p‐*values <.05 were considered to be statistically significant (degrees of freedom [*df*] = 4, 35).

We conducted repetitive water applications (five trials) after the taste stimulation session to determine each neuron subtype's response to the tactile components of tastant application (Figure 1c). We defined pressure‐sensitive neurons (vs. taste‐sensitive neurons) using the following criteria: (a) the neuron responded similarly to the four tastants, (b) the neuron responded to the application of a water rinse, and (c) the neuron responded to repetitive water application. Neurons that met all three criteria were classified as “tactile.” Thereby, eight neurons categorized as pressure‐sensitive neurons were excluded from the present study.

The response duration was calculated as the total time (s) by 100‐msec bin in which the response exceeded the mean pre‐stimulus activity level (threshold: mean + 2 standard deviations [SDs]) during the pre‐stimulus period (2 s). CV was calculated as a ratio of *SD* to the spikes during the taste responses for 6 s and per 1‐s bin for 6 s per five trials.

Each neuron was classified according to the taste stimulus that was the most effective in causing it to respond (best stimulus) (Frank, 1973). In the present study, best stimuli were determined via a statistical comparison of individual neuronal responses to the four tastants (one‐way ANOVA, *F* (4, 35) > 2.64, *p* < .05). For neurons with no significant differences between two preferred tastants, both tastants were considered the best stimulus. For example, NH‐best neurons were defined by the absence of a significant difference between response magnitudes to NaCl and HCl. Individual neurons classified by best‐taste category were confirmed using a correspondence and hierarchical cluster analysis. Pearson's correlation coefficients were calculated to measure the similarity of the net responses to each taste stimulus between neuron pairs. The most similar pair was set in a new cluster, which was measured sequentially to the similarity of another cluster until all neurons were assigned to a cluster. Clustering was performed by the average linkage method using SPSS (IBM).

### 2.5 Unit recordings

Extracellular single‐unit activity was recorded using four glass micropipettes (tip diameter: 1.5 μm, impedance: 4–6 MΩ) glued together to create a 50–100‐μm distance between the contiguous tips. To record from PBN neurons, multibarreled microelectrodes were driven with micro‐step drivers from the cerebellar surface and/or the cerebral surface into the pons. Recording microelectrodes were tilted 16° to the posteroanterior direction or 20° to the anteroposterior direction such that they approached the PBN, avoiding the sinus venosus between the cerebral and cerebellar surfaces. The region explored extensively with microelectrodes was 4.7–5.6 mm anterior and 1.3–2.3 mm lateral to the obex. Action potentials were amplified via a multichannel recording system (DPA‐2008, Dia Medical System, Tokyo, Japan) with a band‐pass of 300 Hz–5 kHz (P‐81, NF Electronic Instruments, Yokohama, Japan). Spiking activity was monitored and digitally sampled. The recorded spikes were isolated from backgound activities in offline with software using a template matching method, as previously described (Forster & Handwerker, 1990). The spike times were translated relative to the time of stimulus onset (resolution: 0.1 msec) and each neuron's activity was displayed as mean peri‐stimulus time course histograms (100‐msec bins) in raster diagrams (1‐msec bin). Custom scripts were written in LabVIEW (National Instrument) to monitor, acquire, sort, and count neuron spiking.

### 2.6 Histologic reconstruction of taste‐sensitive neurons in coronal sections

**FIGURE 2:**
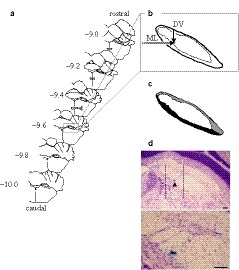
Reconstruction of taste‐sensitive neurons in the pontine parabrachial nucleus (PBN). The locations of dye spots injected via recording microelectrodes were measured on rostrocaudal (RC), mediolateral (ML), and dorsoventral (DV) axes. (a) Numbers on the left of coronal sections indicate caudal distance in mm from the bregma. The caudal border (a gray triangle) of the reticulotegmental nucleus pons lateralis (RtTgL) lies at −9.4 mm. (b) ML indicates the distance from the midline to a dye mark (blue spot), and DV is the distance from the dorsal boundary of the PBN to the dye mark. (c) The PBN was divided into lateral (gray) and medial (black) regions and the brachium conjunctivum (BC) (white). (d) A photomicrograph of PBN sample. Arrowheads indicate dye at the recording sites. Top: a low magnification; Bottom: a high magnification. Each dotted line is a medial or lateral border for the waist area. Schematic drawings in (b) and (c) are the same as that in (d). Scale bar is 0.1 mm

After recording from taste‐sensitive neurons, electrophoresis was used to inject pontamine sky blue (2% in 0.5 M sodium acetate) via each recording microelectrode with application of a 5‐μA DC current for 8 min. At the end of the experiment, the brain was perfused with Ringer's solution, followed by 10% formalin, and stored in a mixture of 30% sucrose and 10% formalin. Serial frozen sections (thickness: 40 μm, coronal plane) were prepared by cryostat (HM505E MICROM, GMI, Minneapolis, USA), maintained at −25°C, and stained with cresyl violet. Images of each section were obtained using a light microscope coupled to a digital camera (Nikon, Tokyo, Japan; 3.34 mega‐pixels; spatial resolution: 30 μm^2^/pixel), which were analyzed with ImageJ (https://imagej.nih.gov/ij/download.html). The position of each neuron (dye mark) was determined using its distance from three reference points (Figure 2): (a) the rostrocaudal (RC) distance from the caudal border (−9.4 mm from the bregma, filled triangle in Figure 2a) of the reticulotegmental nucleus pons lateralis (Paxinos & Watson, 2007), (b) the dorsoventral (DV) distance from the dorsal boundary of the PBN in the coronal plane, and (c) the mediolateral (ML) distance from the midline (Figure 2b). Recording sites were reconstructed in the coronal planes as follows: the recording sites were divided into five groups (−9.1, −9.3, −9.5, −9.7, and −9.9 ± 0.1 mm on the RC axis, respectively); the preparations per group were overlapped based on the outline of the BC using Photoshop (Adobe Inc.), and the dye positions were marked on the coronal plane. To examine the localization of the recording units, the PBN was classified into three regions: a lateral region (gray area), BC (white area), and a medial region (black area, Figure 2c). Furthermore, topographical differences between the waist area (the lateral and medial regions in close vicinity to a constriction of the BC and to the BC itself) and the other region were assessed. The waist area was defined with medial cell invasion (a left dotted line, Figure 2d) to lateral cell invasion (a right dotted line, Figure 2d). A sample recording site (black triangle) was located in the waist area.

### 2.7 Statistical analyses

Numerical values for taste responses are presented as means ± *SE*s. Statistical significance (*p* < .05) was assessed using Student's *t*‐test or one‐way ANOVA with a Tukey's HSD test for comparison of mean values. The similarity of taste profiles between individual neurons was analyzed using hierarchical cluster analyses. The number of neurons in which dye localization marked the recording electrodes was examined via Fisher's exact probability test. These statistical analyses were conducted using SPSS software (IBM).

## 3 RESULTS

### 3.1 The basic characteristics of taste responses in the PBN

**FIGURE 3:**
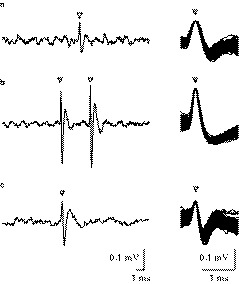
Examples of spike waveforms in single taste‐sensitive neurons. (a–c) single‐unit action potentials are indicated by open arrowheads. The waveforms for the isolated spikes are superimposed (*n* = 100) on right

**FIGURE 4:**
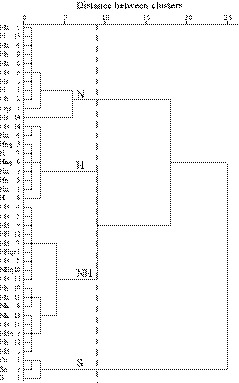
Dendrogram representing the results of hierarchical cluster analysis. Neurons along the ordinate are labeled with the most efficient (capitals) and least efficient (small letters) stimuli. The number sequence for the response profile in descending order of net response in each best stimulus category. The abscissa depicts similarities in taste profiles between individual neurons as the distance on the *x*‐axis. The four clusters indicated by the broken line generally correspond to the results for each best stimulus category. H, H cluster; NH, NH cluster; N, N cluster; S, S cluster

**FIGURE 5:**
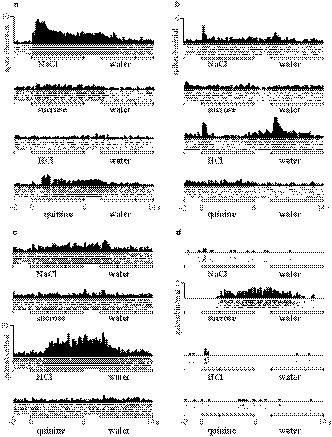
Mean peri‐stimulus time course histograms of representative (a) N‐best, (b) NH‐best, (c) H‐best, and (d) S‐best neurons. The bin‐width is 100 msec. Gray horizontal lines indicate the taste stimulation period (6 s) and the subsequent water rinse period (initial 6 s of 14 s). Spike densities are shown in raster plots (five trials) below the histograms

**FIGURE 6:**
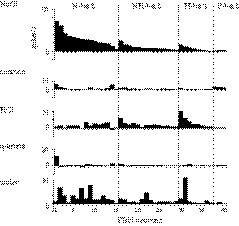
Taste response characteristics. Neurons are aligned in descending order of net response to each preferred best stimulus. The response magnitude for each stimulus is represented as a net response (spikes/s). PBN, pontine parabrachial nucleus

**FIGURE 7:**
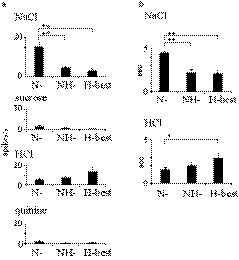
Comparison of taste response properties of N‐best, NH‐best, and H‐best neurons. (a) The mean net response magnitude for NaCl in the N‐best neurons was significantly higher than those in NH‐best and H‐best neurons [*F* (2, 34) = 15.67, *p* < .01, one‐way ANOVA]. (b) The mean response duration for NaCl in the N‐best was significantly longer than those in the NH‐best and H‐best neurons [*F* (2, 34) = 23.8, *p* < .01, one‐way ANOVA]. H‐best neuronal responses to HCl indicated a longer mean response time compared with that of N‐best neurons [*F* (2, 29) = 3.94, *p* < .05, one‐way ANOVA]. *p < .05; **p < .01

A total of 40 taste‐sensitive neurons were recorded from in 31 animals. Single‐unit activity was recorded in all cases. Single‐unit activity was recorded in all recordings (Figure 3). Neurons were classified into the following best stimulus categories: N‐best (*n* = 15), NH‐best (*n* = 14), H‐best (*n* = 8), and S‐best (*n* = 3) neurons in the PBN (Table 1). To assess the validity of each best stimulus category, taste‐sensitive neurons were further analyzed via a hierarchical cluster analysis using their net responses (Figure 4). This analysis indicated four groups or functional clusters: N, H, NH, and S. The results of this cluster analysis corresponded to the classifications resulting from the best stimulus analysis (33/40, 83%). Two‐thirds of N‐best neurons (10/15, 67%) were assigned to the N cluster, while all H‐best neurons (8/8, 100%) were successfully assigned to the H cluster. Most of the NH‐best neurons (12/14, 86%) were assigned to the NH cluster, while all S‐best neurons (3/3, 100%) were assigned to the S cluster. There were some disagreements between these analyses, with five N‐best neurons assigned to the NH cluster. In these neurons, net responses to NaCl were significantly larger than those to HCl [#Nh 40, *F* = 49.00: #Nh 11, *F* = 80.89, #Nh 8, *F* = 38.01: #Nh 13, *F* = 65.30: #Nh 12, *F* = 27.56, *df* (4, 35), *p* < .01, one‐way ANOVA: *p* < .01, post hoc test]. Therefore, we considered these to be an intermediate type of N‐best neurons. Peri‐stimulus time histograms for the N‐best, NH‐best, H‐best, and S‐best neurons are represented in Figure 5. Recording neurons were aligned in descending net response order for each best stimulus (Figure 6). The net responses to NaCl among N‐best neurons appear to be larger than those for NH‐best neurons (Figures 5a and b and 6). As shown in Figure 5b, the neuronal activities saliently increased during water rinses after HCl stimulations, and somewhat after NaCl stimulations. The responses after tastants have previously been reported for rat chorda tympani (CT) as the off (water)‐responses (Beidler 1967; DeSimone, Callaham, & Heck, 1995; Formaker, Stapleton, Roper, & Frank, 2004; Yamamoto & Kawamura, 1974). In order to clarify the response characteristics, we compared net responses between N‐best, NH‐best, and H‐best neurons. Net responses to NaCl (15.2 ± 2.3 spikes/s) among N‐best neurons were significantly larger than among NH‐best neurons [Figure 7a; 4.5 ± 0.8 spikes/s, *F* (2, 34) = 15.67, *p* < .01, one‐way ANOVA]. As shown in Figure 7b, the NaCl response duration (4.5 ± 0.2 s) of N‐best neurons was significantly longer than for NH‐best neurons [2.2 ± 0.3 s; *F* (2, 34) = 23.8, *p* < .01, one‐way ANOVA].

**TABLE 1:** Mean net responses for taste‐sensitive neurons classified by stimuli

	NaCl	HCl	Sucrose	Quinine	Water (spikes/s)	*n*
*N*‐best	**15.2 ± 2.3**	2.7 ± 0.6	1.4 ± 0.5	0.9 ± 0.8	8.2 ± 1.7	15
NH‐best	4.5 ± 0.8	3.8 ± 0.7	0.4 ± 0.3	0.3 ± 0.1	3.5 ± 1.1	14
H‐best	3.1 ± 0.9	6.9 ± 2.3	0.2 ± 0.2	0.4 ± 0.2	5.7 ± 4.2	8
S‐best	0.8	0.3	2.8	0.2	0.6	3

Note

Numbers (mean ± *SE*) in columns with tastant names indicate net taste response (spikes/s) to each taste stimulus (spikes during water application subtracted). Firing rates for each tastant are compared between the four stimulus categories for neurons in each column. Bold values indicate significantly larger spike activity compared with the other three stimulus categories. One‐way ANOVA (*p* < .01) used for hypothesis testing, followed by paired comparisons with Tukey's HSD test (*p* < .05). *n* = number of neurons.

**FIGURE 8:**
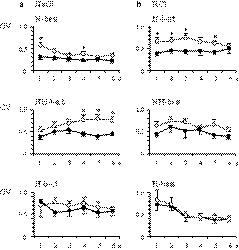
Comparison of coefficient of variation (CV) between the rNST and the PBN neurons. (a) Mean CV for NaCl stimulation by 1‐s bin for 6 s. (b) Mean CV for HCl stimulation. Open circle and gray line: the rNST. Filled circle and black line: the PBN. The mean CVs in the PBN neurons were significantly smaller in the N‐best (*p < .05, *t*‐test, *df* = 36.0) and the NH‐best neurons (*p < .05, *t*‐test, *df* = 37.0)

NaCl response properties for N‐best neurons were distinct from those for NH‐best neurons in the PBN. We analyzed the differences between neuron performance (net responses, response durations, and CV) in the PBN in the current study and those in the rNST, as reported in our previous work (Yokota et al., 2014). There were no differences across taste stimuli between the PBN and the rNST in either the net responses or response duration (*p* > .05, *t*‐test). However, the mean CV for the N‐best and the NH‐best neurons in the PBN was significantly smaller than those in the rNST for some time course in 1‐s bin during NaCl stimulations (1 and 4 s in the N‐best, 4–6 s in the NH‐best in Figure 8a, *p* < .05, *t*‐test). The N‐best neurons in the PBN indicated the smaller CVs for HCl stimulations (1–3 and 5 s in Figure 8b, *p* < .05, *t*‐test). Here, the smaller CV indicates that neuronal activities were less variable per trials. The mean net responses in 1‐s bin were similar between the rNST and the PBN (*p* > .05, *t*‐test). The significant difference for 6 s was not found for both the net responses and the CV (*p* > .05, *t*‐test).

### 3.2 Histological localization of taste‐sensitive neurons classified by stimulus response

**FIGURE 9:**
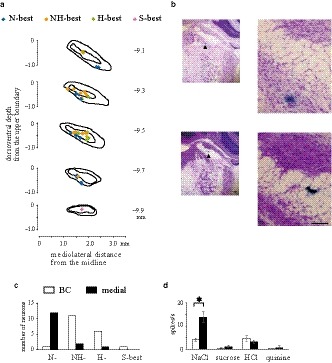
Topographical distribution of pontine parabrachial nucleus (PBN) neurons for each preferred stimulus category. Individual neurons (diamonds) classified into N‐best (blue), NH‐best (orange), H‐best (yellow green), and S‐best (pink) categories. (a) Recording sites were reconstructed in the coronal plane. (b) Photomicrographs of recording sites in the PBN. Arrowheads indicate exemplar dye marks ejected from recording electrodes. Top: N‐best neuron; Bottom: NH‐best neurons. Scale bar is 0.1 mm. a low (left) and a high (right) magnification. (c) Most N‐best neurons were located in the medial region of the PBN, while NH‐best and H‐best neurons were mainly found in the brachium conjunctivum (BC). There was a significant difference between the proportions of N‐best and other neurons (*p* < .01, Fisher's exact probability test). (d) Mean net responses to NaCl in the medial region were significantly greater than that in the BC (*p < .01, *t*‐test, *df* = 17.4)

In order to examine the topographical distribution of taste‐sensitive neurons on the PBN, dye marks ejected from recording electrodes were reconstructed in coronal sections. A total of 34 recording sites were colored and superimposed onto coronal sections 0.2 mm in thickness (Figure 9a). The remaining six recording sites remained unidentified due to dye ejection failure. Taste‐sensitive neurons were not found in the lateral region. The photomicrographs further indicated representative dye marks for N‐best (the top in Figure 9b) and NH‐best (the bottom in Figure 9b) neurons. Most N‐best neurons were observed in the medial regions (12/13 neurons, 92% in Figure 9c), while NH‐best and H‐best neurons were more often located in the BC (NH‐best, 11/13 neurons, 85%; H‐best, 6/7 neurons, 86%). The number of recording sites significantly differed between the N‐best neurons (12 neurons in the medial and one neuron in the BC) and others (three neurons in the medial and 18 neurons in the BC) (*p* < .01, Fisher's exact probability test). The mean response magnitude for NaCl stimulation was larger in the medial region (13.9 ± 2.4 spikes/s, *n* = 15) than in the BC (4.1 ± 0.8 spikes/s, *n* = 19) (Figure 9d; *p* < .01, *t*‐test, *df* = 17.4). These results may be explained by a larger response magnitude in N‐best neurons compared with the NH‐best and H‐best neurons (Figure 7a).

On the RC axis (Figure 9a), the distributions of these three neuronal subsets overlapped considerably. Most taste‐sensitive neurons (26/34 neurons, 76%) were found in the caudal half (the RC < −9.30 mm from the bregma), when a range of PBNs was determined from −8.64 to −9.96 mm from the bregma on the RC axis (Paxinos & Watson, 2007). On the DV axis (Figure 9a), the N‐best neurons were located more ventrally than NH‐best and H‐best neurons.

**FIGURE 10:**
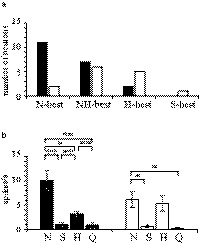
Characteristics of taste responses in the waist area. (a) The number of neurons was counted in the waist area (black column) and non‐waist area (white column). (b) The mean net response to tastants in the waist (black column) and non‐waist areas (white column). One‐way ANOVA (*p* < .01) was followed by paired comparisons with Tukey's HSD test (*p < .05, **p < .01)

A medial and a lateral end of the constricted BC were measured in histological preparations (Figure 1d). When an ML value was between the medial and lateral end of the preparation, its dye spots were counted as in the waist area. The remaining one (an S‐best) was not measured, because its waist area was not clear in the most caudal preparation (RC = −9.9 mm in Figure 9a). Two‐thirds of taste‐sensitive neurons (21/34 neurons, 62%) were located in the waist area with the remaining neurons (13/34, 38%) outside of the waist area. The N‐best (11/13, 85%), NH‐best (8/13, 62%), and H‐best (2/7, 29%) neurons were located in the waist area (Figure 10a). The location of N‐best neurons was significantly biased toward the waist area when compared with the other neurons (*p* < .05, Fisher's exact probability test). Mean net responses to NaCl in the waist area were prominent among the tastants [black columns in Figure 10b *F* (3, 76) = 14.45, *p* < .01, one‐way ANOVA; **p < .01 or * *p* < .05, post hoc test]. There were, however, no differences between N‐best and H‐best neurons’ responses in the non‐waist area [white columns in Figure 10b *F* (3, 52) = 7.61, *p* < .01, one‐way ANOVA; *p* > .05, post hoc test].

## 4 DISCUSSION

The mean net response for N‐best neurons to NaCl in the present study was significantly greater than that for NH‐best and H‐best neurons. The mean response duration of N‐best neurons to NaCl was also significantly longer than that of NH‐best and H‐best neurons. Most of the N‐best neurons were located medially, whereas NH‐best and H‐best neurons were located in the BC. N‐best neurons were clearly distinguished from NH‐best and H‐best neurons in terms of both their response properties and physical distributions.

### 4.1 Comparison of PBN and rNST cellular taste properties

Most of the taste‐sensitive neurons encountered in the present study were classified into at least one of three different groups (the N‐best, NH‐best, and H‐best) according to their net response similarities. The mean net response to NaCl for the N‐best neurons was significantly larger than that for the NH‐best and H‐best neurons. Furthermore, the mean response duration for N‐best neurons to NaCl was significantly longer than that for NH‐best and H‐best neurons.

Differences between the N‐best (i.e., the greater responses with persistent activity) and NH‐best (smaller responses with phasic activity) were also observed among rNST neurons in a previous study by our group (Yokota et al., 2014). The characteristics of the N‐best and NH‐best neurons identified here appear to be consistent between the rNST and PBN in terms of both net responsivity and response duration. Two related reviews have reported differing results with regard to the amplification of taste responses in the rNST and PBN—in one, an increase from the rNST to the PBN of only ~10% (Di Lorenzo & Monroe, 1997; Di Lorenzo, Platt, & Victor, 2009) was noted, while in the other, the taste responses in the PBN were found to be only half as large as those in the rNST (Scott & Small, 2009). This discrepancy may be due to differing experimental methods and/or the recording parameters used/neurons selected for recording.

The subset of PBN neurons (the N‐best and the NH‐best neurons) represented the smaller CV for certain times in 1‐s bin than those of the rNST. Fluctuations of neuronal activities in the rNST may be suppressed in the PBN. Convergent inputs on sensory pathways reduced variability in spike generations (Carr, Heiligenberg, & Rose, 1986; Xu‐Friedman & Regehr, 2005). The neuronal activities in the PBN may be stabilized by the convergent inputs compared with those in the rNST.

The degree of correspondence between the best stimulus category and PBN cluster analysis (83%) in the current study was similar to the rNST (89%) (Yokota et al., 2014). The number of neurons with responses to second‐order stimuli, in addition to best stimuli responses, in the PBN (24/40 neurons, 60%) tended to be greater than that in the rNST (37/73 neurons, 51%). This result may suggest some convergence of neurons with different taste properties on the PBN. Convergence to PBN has been reported for taste and tactile, taste and visceral, and oral receptive fields (Halsell & Travers, 1997; Karimnamazi et al., 2002; Travers & Geran, 2009). Further studies may address the contributions of the convergence to taste discrimination.

### 4.2 Topographical representations in the PBN

In the present study, a topographical distribution of taste‐sensitive neurons preferentially activated by different stimuli was revealed along the DV and ML axes. Most of the N‐best neurons assessed here were located in the medial region, and more particularly in the waist area. However, NH‐best and H‐best neurons were primarily located in the BC.

Taste‐sensitive neurons have been reported in the medial region and dorsal edge of the BC and within the BC by electrophysiological studies (Geran & Travers, 2009; Halsell & Frank, 1991; Norgren & Pfaffmann, 1975; Ogawa et al., 1984). Previous studies conducted on rats and hamsters have not reported the distribution of N‐best and H‐best neurons in detail except that these neurons tend to occur in the caudal‐ventral and the rostral‐lateral regions, respectively. (Van Buskirk & Smith, 1981; Ogawa et al., 1984). These observations are consistent with the topography of N‐best neurons and the ensembles of NH‐best and H‐best neurons in the current study. The net response magnitudes for NaCl were correlated to the DV, with significantly larger mean net response magnitudes in the medial region than in the BC in the present study. When PBN neurons were antidromically activated by electrical stimulation in the third taste relay—the parvicellular division of the thalamic posteromedial ventral nucleus (VPMpc) in rats—the mean net response for NaCl in the medial region was significantly larger than that in the lateral region (Ogawa et al., 1984). Previous study has shown that c‐fos cells evoked by NaCl and reduced by amiloride, an epithelial Na^+^ channel blocker, are preferentially located in the central medial subnucleus (Yamamoto et al., 1993), which corresponds to the medial region in our study. These results indicate that the medial region transmits NaCl information more robustly to the thalamus. In mice, sucrose responses in the medial region were significantly larger than in the BC, but not in the lateral region (Tokita & Boughter, 2016). In mice, taste‐sensitive neurons frequently respond to sucrose, with sweet tasting stimuli proving most effective for TRCs at the CT, rNST, and PBN (McCaughey, 2007; Tokita, Yamamoto, & Boughter, 2012; Yoshida, Yasumatsu, Shigemura, & Ninomiya, 2006), although mouse strains differed in sweetness responses at rNST (McCaughey, 2007). This parallels NaCl responsivity in rats because sodium salts are the most effective stimuli for rats (Halsell & Travers, 1997; Monroe & Di Lorenzo, 1995; Ogawa, Sato, & Yamashita, 1968; Verhagen, Giza, & Scott, 2003; Yamamoto et al., 1985). In spite of differences between responsivity to spices and tastants, the medial region in the PBN may play an important role in informing the rest of the brain about the nutrient content of a given food, thus maintaining a vital function for the species.

We did not record from the taste‐sensitive neurons in the lateral region of the PBN in the present study. One reason for this was the small size of the subnucleus of taste‐sensitive neurons in the lateral region (Halsell & Frank, 1991). Furthermore, the taste‐sensitive neurons in this lateral region are less successfully recorded than those in the BC or the medial region, as evidenced previously (Geran & Travers, 2009). However, taste‐sensitive neurons have been observed in the lateral region (Halsell & Frank, 1991; Halsell & Travers, 1997; Ogawa, Hayama, & Ito, 1987; Tokita & Boughter, 2016). The present study had a limitation for the topographical distribution due to lack of recordings in the lateral region.

### 4.3 Oral representation in the DV axis

Receptive fields for the tongue appear to be related to the localization of taste‐sensitive neurons in the PBN. Taste responses to anterior tongue stimulation have been observed in the medial region and for the posterior tongue, more dorsally to and within the BC (Norgren & Pfaffmann, 1975). The taste‐sensitive neurons with the receptive field at the anterior tongue were located in the ventral lateral subnucleus as well the central medial subnucleus (Halsell & Travers, 1997). Of relevance, the anterior tongue is innervated by the CT, while the posterior tongue is primarily innervated by the glossopharyngeal nerve (IX). In the rats, the CT vigorously responds to NaCl and HCl (Frank, Contreras, & Hettinger, 1983), while the IX is responsive to quinine and HCl (Frank, 1991; Sako, Harada, & Yamamoto, 2000). Taken together, it is likely that NaCl responses among medial N‐best neurons may originate from the CT, while HCl responses for NH‐best and H‐best neurons in the BC may arise from the IX. In the CT, the taste responses to NaCl in the N‐best fibers were markedly reduced by oral applications of amiloride, whereas the taste responses to NaCl in the H‐best fibers were not reduced by amiloride (Hettinger & Frank, 1990; Ninomiya & Funakoshi, 1988). On the other hand, taste responses to NaCl in the IX were exclusively amiloride‐insensitive (Formaker & Hill, 1991; Kitada, Mitoh, & Hill, 1998). The c‐fos cells evoked by NaCl stimulations were observed in the central medial and the external lateral subnuclei in the PBN, and the c‐fos cells in the central medial were reduced by amiloride (Tokita, Shimura, Nakamura, Inoue, & Yamamoto, 2007; Yamamoto et al., 1993). Furthermore, genetic engineering studies revealed that amiloride‐sensitive and ‐insensitive TRCs responded to the palatable salt (low concentrations) and the aversive salt (high concentrations), respectively (Chandrashekar et al., 2010; Oka, Butnaru, von Buchholtz, Ryba, & Zuker, 2013). These results suggest that the medial region in the PBN (i.e., the N‐best neurons) is a part of the palatable‐salt pathway originating from the amiloride‐sensitive TRCs. On the other hand, information for the aversive salt may be transmitted to the NH‐best and H‐best neurons in the BC. The taste‐topic map in the PBN appears to represent the aversive‐salt and palatable‐salt pathways dorsoventrally.

### 4.4 Functional role of the waist area

Most of the N‐best neurons were located in the waist area and were more in number than other, taste‐selective subtypes of neurons. A previous study reported that N‐best and H‐best neurons are located within the waist area and its vicinity (Halsell & Travers, 1997). In the present study, we found that H‐best neurons were primarily located outside of the waist area. This discrepancy may have resulted from different definitions of neurons based on their selective responsivity to differing stimuli (e.g., how we distinguished between H‐best and NH‐best neurons).

The functional roles of the PBN have been investigated in relationship to the external lateral, external medial, dorsal lateral, ventral lateral and central medial subnucleus surrounding the BC. Different patterns of c‐Fos immunoreactivity have been demonstrated in the distinct subnuclei of the PBN, likely involved in positive or negative hedonic responses (Gaykema et al., 2009; Hashimoto, Obata, & Ogawa, 2009; Kobashi, Ichikawa, Sugimoto, & Adachi, 1993; Yamamoto, Shimura, Sakai, & Ozaki, 1994). Anterograde labeled rNST terminals located in the medial region of the rostral PBN and in the lateral‐medial regions have also been associated with waist area neurons in the caudal PBN in rats (Hermann, Kohlerman, & Rogers, 1983). Retrograde labeling of the VPMpc has revealed the projection of neurons in the PBN to the external lateral subnucleus and the medial and ventral lateral subnuclei (the waist area) in mice (Hashimoto et al., 2009; Tokita, Inoue, & Boughter, 2010). These results indicate that the waist area plays an important role for transmission of sensory information from the rNST to the VPMpc. Taken together, NaCl taste information may be sent mainly from the waist area to the VPMpc. The topographical distribution of neurons in the PBN may further contribute to the maintenance of accurate NaCl‐related information transmission.

Taste information for sodium salt may be separately conserved between N‐best and NH‐best neurons from the rNST to PBN. The present study could not investigate the neuronal networks between the medial region and the BC in the PBN, and between the PBN and higher order nuclei (probably the VPMpc and amygdala). Further studies could clarify the brain structures and the relationships underlying taste discrimination, which is needed to maintain an animal's wellness.

## CONFLICT OF INTEREST

No conflict of interest, financial or otherwise is declared by the author(s).

## AUTHOR CONTRIBUTIONS

T.Y., N.K., and K.H. conceived and designed the research; T.Y. and T.Mo. performed experiments; T.Y., T.Mo., and T.Ma. analyzed data; T.Y., N.K., and K.H. interpreted the results of experiments; T.Y. and N.K. prepared figures; T.Y. and N.K. drafted the manuscript.
